# Association of Testosterone With Lean Soft Tissue and Handgrip Strength Across Middle‐Aged Men

**DOI:** 10.1002/jcsm.70329

**Published:** 2026-07-07

**Authors:** Konstantinos Prokopidis, Stefano Cacciatore, Joseph McLean, Paolo Piaggi, Carla M. Prado, John A. Batsis, Mathias Schlögl

**Affiliations:** ^1^ Department of Musculoskeletal Ageing and Science, Institute of Life Course and Medical Sciences University of Liverpool Liverpool UK; ^2^ Department of Physiology and Aging, College of Medicine University of Florida Gainesville Florida USA; ^3^ Androlabs London UK; ^4^ Department of Information Engineering University of Pisa Pisa Italy; ^5^ Department of Agricultural, Food and Nutritional Science University of Alberta Edmonton Alberta Canada; ^6^ Division of Geriatric Medicine, School of Medicine, and Department of Nutrition, Gillings School of Global Public Health University of North Carolina at Chapel Hill Chapel Hill North Carolina USA; ^7^ Geriatric Medicine Clinic Barmelweid AG Barmelweid Switzerland

**Keywords:** ageing, body composition, muscle mass, muscle strength, sarcopenia, testosterone

## Abstract

**Background:**

Testosterone declines by 0.4%–2% annually after the age of 30 and is potentially linked with muscle mass and strength. This study examined how testosterone levels are associated with handgrip strength (HGS) and appendicular lean soft tissue index (ALSTI) in men aged 40–49 and 50–59 years.

**Methods:**

Data were sourced from the National Health and Nutrition Examination Survey (NHANES) cycles (2011–2014). The median values of each cohort (378.2 and 378.6 ng/dL for those aged 40–49 and 50–59 years of age, respectively) and the European Association of Urology guidelines were used to define higher serum testosterone, HGS and ALSTI. Linear and logistic regressions assessed associations between higher/lower serum testosterone with higher/lower HGS and ALSTI for each age group.

**Results:**

In 1001 men aged 40–59 years, higher total testosterone levels were positively associated with higher HGS, with stronger links in the 50‐ to 59‐year‐olds (*b* = 1.35, 95% confidence interval [CI] 0.08–2.62, *p* < 0.01). In this group, higher testosterone was linked to increased odds ratio (OR) of having higher HGS (OR: 1.73, 95% CI 1.17–2.55, *p* < 0.01). Per European Association of Urology guidelines, men with testosterone levels above deficiency (≥ 230 ng/dL) had higher odds of increased ALSTI versus those with deficiency (OR: 3.31, 95% CI 1.42–7.74, *p* < 0.01), while normal testosterone (> 346 ng/dL) versus deficiency showed a significant albeit weaker association (OR: 2.48, 95% CI 1.35–4.57, *p* < 0.01). Linear regression confirmed an ALSTI increase above deficiency (*b* = 0.20, 95% CI 0.05–0.34, *p* < 0.01); however, normal versus suspected deficiency for total testosterone was linked to a small but potentially minor clinically significant difference with ALSTI in the whole cohort (*b* = 0.10, 95% CI 0.00–0.19, *p* = 0.049). Normal testosterone was linked to higher HGS overall (OR: 1.37, 95% CI 1.05–1.80, *p* = 0.02) and in men aged 50–59 years (OR: 1.53, 95% CI 1.03–2.28, *p* = 0.03).

**Conclusions:**

Normal and higher than deficiency testosterone levels in men aged 40–59, particularly 50‐ to 59‐year‐olds, are associated with higher HGS and ALSTI compared with those with deficient testosterone concentrations.

## 1 Introduction

Skeletal muscle health is a key determinant of functional independence in ageing populations. Sarcopenia, the age‐related loss of muscle mass and strength, is a phenomenon emerging in the 5th decade of life [1], influencing critical measures such as handgrip strength (HGS) and skeletal muscle mass. High HGS is widely regarded as a key indicator of upper body muscle strength and has been associated with lower mortality and improved physical function [2]. Similarly, appendicular lean soft tissue (ALST; the sum of fat‐ and bone‐free mass in the arms and legs) has been shown to be another surrogate marker of muscle health. Both HGS and ALST are linked to quality of life and can serve as predictive factors for adverse health outcomes, including disability, frailty and the risk of falls [3, 4].

Multiple factors may accelerate the onset and clinical impact of sarcopenia, including health status, anabolic resistance and hormonal imbalances. Testosterone, a key anabolic hormone, is suggested to play a pivotal role in maintaining muscle mass, strength and overall physical function, particularly in older adults [5]. As an anabolic steroid hormone, testosterone influences muscle protein synthesis (MPS) [6], promoting muscle growth and repair [7, 8]. The bulk of high‐quality evidence suggests that, on average, testosterone levels decrease by 0.4%–2% each year after the age of 30 [9]. In some men, this can lead to a clinical deficiency in testosterone which, when accompanied by specific symptoms of testosterone deficiency, leads to a condition known as hypogonadism [10]. The European Association of Urology (EAU) guidelines outline a repeated total testosterone reading of below 346 ng/dL (12 nmol/L) as biochemical evidence of hypogonadism [11].

The age‐related decline in testosterone levels has been implicated in numerous age‐related conditions, including sarcopenia and frailty [12, 13] as it has been associated with a reduction in lean mass and HGS [14, 15]. However, much of the available clinical and functional evidence linking testosterone to muscle health derives from studies conducted in older adults or late‐life populations, in whom sarcopenia, multimorbidity and functional impairment are often already established [16]. Consequently, less is known about when during midlife declining testosterone levels begin to translate into meaningful differences in muscle mass and strength. In particular, the timeframe between 40 and 60 years represents a critical yet understudied transition period during which anabolic resistance and testosterone decline accelerate, but overt sarcopenia and disability are often not yet clinically apparent. Treating midlife as a homogeneous phase may therefore obscure early endocrine–muscle relationships that precede established functional decline, limiting the ability to identify individuals at risk at a stage when muscle function is still largely preserved and potentially modifiable.

Therefore, this study examines how differences in testosterone levels during midlife relate to clinically interpretable variations in muscle strength and LST, with particular attention to guideline‐relevant hormonal thresholds. Using data from the National Health and Nutrition Examination Survey (NHANES), we investigated associations between total testosterone levels, HGS and ALST in men aged 40–49 and 50–59 years, anchoring testosterone categories to established clinical guideline cutoffs to enhance clinical interpretability and early risk stratification.

## 2 Methods

Data on serum total testosterone, ALST and HGS were obtained from the 2011–2012 and 2013–2014 NHANES cycles, in which these measures were concurrently available. NHANES, a series of cross‐sectional surveys conducted by the National Center for Health Statistics (NCHS) under the US Centers for Disease Control and Prevention (CDC), gathers data through interviews, physical exams and laboratory assessments. The NHANES database is publicly available for access via the NCHS website. The study protocol was approved by the NCHS Ethics Review Committee, and all participants provided informed consent.

**FIGURE 1:**
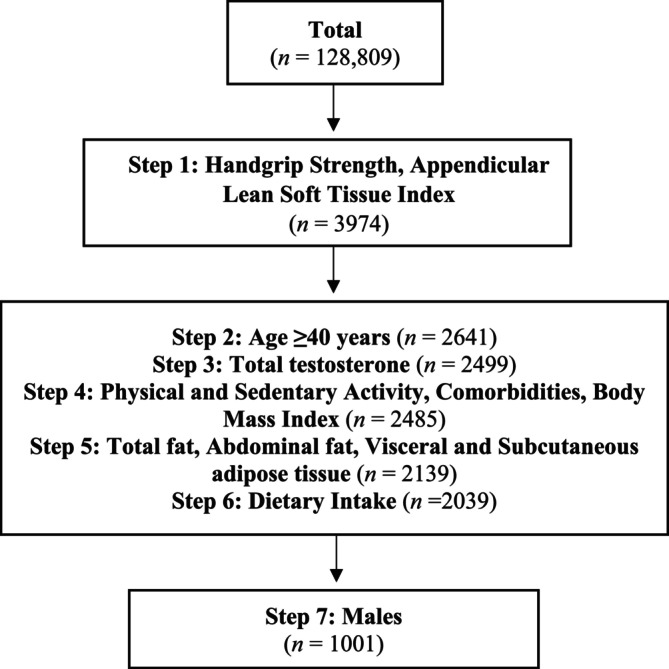
Flowchart of the sample selection.

There was a total of 128 809 participants screened, from which 2641 had data for HGS, ALST, and 40 years as minimum age, concomitantly. Individuals without data on total testosterone, physical activity, body mass index (BMI), comorbidities and body composition were filtered, leaving 2139 adults. Adults with data on dietary intake were subsequently filtered (*n* = 2039), and finally, data was filtered based on men, for which our final cohort consisted of 1001 subjects. The restriction to men aged 40–59 years was intentional and hypothesis‐driven, aiming to examine endocrine–muscle associations during midlife, before factors such as multimorbidity, survival bias and testosterone replacement therapy (TRT) become increasingly prevalent and may obscure underlying biological relationships in older populations. A flowchart illustrating the selected sample size can be found in Figure 1.

### 2.1 Baseline Characteristics

Data on race (Mexican, non‐Hispanic White, non‐Hispanic Black, Hispanic and others), education (college or above, college or associate degree, high school and others), comorbidities (arthritis, cancer, diabetes) and physical and sedentary activity (moderate, sedentary) were collected via self‐administered questionnaires. Dietary intake (energy, protein, carbohydrate, fibre, fat intake) was collected based on two 24‐h recalls, for which the average value was obtained. BMI was derived as weight divided by height squared. Serum total cholesterol and serum high‐density lipoprotein cholesterol (HDL‐C) levels were measured enzymatically.

### 2.2 Assessment of Total Testosterone, Handgrip Strength and Lean Soft Tissue

Total serum testosterone levels from overnight fasted samples were measured using the isotope dilution‐liquid chromatography–tandem mass spectrometry (ID–LC–MS/MS) technique. There is no universal consensus among endocrinology and urology guidelines on the definition of testosterone deficiency, with deficiency cut‐off values ranging from 230 to 346 ng/dL, depending on the specific guideline body [17], although normal levels range from 450 to 600 ng/dL [18]. The present analysis defined lower levels of testosterone as the bottom 50th percentile of the study population, which equated to a mean testosterone value of 378.2 and 378.6 ng/dL for those aged 40–49 and 50–59 years of age, respectively. Higher total testosterone, HGS and ALST were defined as the median of the cohort based on age (40–49 and 50–59 years separately). Total testosterone was further categorised as suspected deficiency, deficient and normal, based on the EAU guidelines (suspected deficiency [11]) and the Society of Endocrinology guidelines (highly deficient [19]):

- Normal total testosterone → ≥ 346 ng/dL
- Total testosterone suspected deficiency → < 346 ng/dL
- Total testosterone deficiency → < 230 ng/dL
- Above deficient total testosterone → ≥ 230 ng/dL

Sex hormone–binding globulin (SHBG) concentrations were measured using a chemiluminescent immunoassay.

HGS was evaluated through three attempts for each arm, using a hand dynamometer. A qualified trainer described and showed the procedure to the participant. The instructor then modified the dynamometer's grip size to fit the participant's hand and instructed them to perform a trial squeeze. This trial ensured the participant understood the process and the grip size was correctly set. Following practice, the participant was directed to grip the dynamometer with one hand as forcefully as possible, exhaling during the squeeze to prevent intra‐thoracic pressure buildup. The process was then repeated with the other hand. Each hand underwent three trials, with hands alternated and a 60‐s rest between measurements for the same hand. The grip test was conducted standing, unless the participant had physical limitations. The average of the highest HGS value from each hand was used in this study.

Whole body scans were administered to eligible survey participants using dual X‐ray absorptiometry (DXA) (Hologic QDR‐4500A fan‐beam densitometer). ALST was estimated using a prediction equation that incorporated the lean mass of both the lower and upper limbs (left and right arms and legs). ALST was then divided by height squared (kg/m^2^) to estimate ALST index (ALSTI).

### 2.3 Statistical Analysis

Characteristics of the included participants were compared using student's t‐test for continuous variables and a chi‐squared test for categorical variables between age groups. All data were combined into a single dataset following weighting principles outlined by the NHANES. A linear regression analysis was employed to evaluate the association between higher total serum testosterone levels and HGS and ALSTI as continuous variables. Initially, an unadjusted Model 1 was employed, while Model 2 included potential covariates such as age, sex, body mass index, race and education. In Model 3, additional covariates, including arthritis, diabetes and cancer, were incorporated. Additionally, a binary logistic regression model was used to estimate the odds ratio (OR) and 95% confidence interval (95% CI) of higher total testosterone levels with higher HGS or ALST. The covariates included in the univariate and multivariate models were consistent with those used in the linear regression analysis. An interaction between higher total testosterone levels and age (50–59 and 40–49 years) was employed in the full model prior to age‐specific analyses, controlling for relevant covariates. All analyses were also performed separately for adults aged 40–49 and 50–59 years to assess potential age‐related differences. Moreover, linear and logistic regressions were conducted based on normal versus suspected deficiency total testosterone, normal versus deficient total testosterone, and above deficient total testosterone versus deficient total testosterone, according to the EAU guidelines and the Society of Endocrinology. As a sensitivity analysis, all fully adjusted linear and logistic regression models were repeated in the subsample of participants with available SHBG measurements, with SHBG included as an additional covariate. A *p* value of 0.05 was considered statistically significant for all tests, using IBM SPSS version 29.0.

## 3 Results

The overall sample size consisted of 1001 adults aged 40–59 years (Figure 1). Total testosterone was similar between age groups (*p* = 0.77); however, those aged 50–59 years had lower HGS (*p* < 0.01) and ALSTI (*p* = 0.03) versus 40‐ to 49‐year‐olds. Adults in the 50‐ to 59‐year‐old group had a higher prevalence of arthritis, diabetes and cancer than adults aged 40–49 years. In addition, men aged 40–49 years had higher serum albumin, haemoglobin and total cholesterol, but lower HDL‐C levels compared with those 50–59 years. A table of characteristics for the entire cohort is presented in Table 1 and according to total testosterone status for each age group in Table 2.

**TABLE 1:** Characteristics of the entire cohort and based on age. Data are expressed as mean (standard deviation).

Outcomes	Entire cohort	Aged 40–49 years	Aged 50–59 years	*p*
Sample size (*n*)	1001	516	485	—
Age (years)	49.3 (5.8)	44.4 (2.9)	54.5 (2.9)	< 0.01*
Race, *n* (%)	156 (16)	89 (17)	67 (14)	0.33
Mexican American	207 (21)	99 (19)	108 (22)	
Other Hispanic	421 (42)	209 (41)	212 (44)	
Non–Hispanic White	79 (8)	44 (9)	35 (7)	
Non–Hispanic Black	138 (14)	75 (15)	63 (13)	
Other race				
Education, *n* (%)	283 (28)	146 (28)	137 (28)	0.2
College or above	284 (28)	146 (28)	138 (29)	
College or associate degree	223 (23)	126 (24)	97 (20)	
High school	212 (21)	98 (19)	113 (23)	
Other (below high school)				
Body mass index (kg/m^2^)	28.9 (5.7)	29.0 (5.6)	28.8 (5.7)	0.59
Arthritis, *n* (%)	163 (16)	62 (12)	101 (21)	< 0.01*
Cancer, *n* (%)	46 (5)	9 (2)	37 (8)	< 0.01*
Diabetes, *n* (%)	127 (13)	51 (10)	76 (16)	< 0.01*
Moderate activity (min/week)	31.5 (60.3)	31.2 (58.3)	31.9 (62.3)	0.87
Sedentary activity (min/day)	388.4 (201.3)	390.8 (203.7)	385.9 (198.9)	0.7
Energy intake (kcal/day)	2308 (912)	2341 (926)	2272 (896)	0.24
Protein intake (g/day)	92.6 (39.8)	93.2 (39.1)	91.9 (40.5)	0.6
Carbohydrate intake (g/day)	273.4 (119.1)	277.6 (121.1)	268.9 (116.9)	0.25
Fibre intake (g/day)	19.1 (41.3)	18.7 (10.8)	19.6 (11.6)	0.16
Fat intake (g/day)	86.6 (41.3)	87.9 (41.1)	85.3 (41.4)	0.33
Total cholesterol (mg/dL)	198.2 (42.9)	201.2 (42.3)	195.0 (43.3)	0.02*
High‐density lipoprotein cholesterol (mg/dL)	47.4 (13.4)	46.6 (12.4)	48.3 (14.3)	0.04*
Haemoglobin (mg/L)	14.9 (1.3)	15.1 (1.3)	14.8 (1.4)	< 0.01*
Serum albumin (g/L)	4.33 (0.32)	4.38 (0.29)	4.28 (0.36)	< 0.01*
Total testosterone (ng/dL)	401.6 (167.7)	400.1 (164.5)	403.2 (171.1)	0.77
Total testosterone cut‐off (ng/dL)	378.39	378.2	378.64	—
Sex hormone binding globulin (nmol/L)	41.75 (20.92);	37.98 (18.11);	44.64 (23.19);	< 0.01*
	*n* = 479	*n* = 252	*n* = 227	
Handgrip strength (kg)	44.9 (7.2)	46.8 (6.9)	43.4 (7.1)	< 0.01*
Appendicular lean soft tissue index (kg/m^2^)	8.87 (1.39)	8.96 (1.38)	8.77 (1.38)	0.03*

* Indicates significance.

**TABLE 2:** Characteristics of the cohort based on age and total testosterone levels of the cohort. Data are expressed as mean (standard deviation).

	Aged 40–49 years				Aged 50–59 years			
Outcomes	All	Lower testosterone	Higher testosterone	*p*	All	Lower testosterone	Higher testosterone	*p*
Sample size (*n*)	516	258	258	—	485	243	242	—
Age (years)	44.4	44.3 (2.9)	44.5 (2.8)	0.6	54.5 (2.9)	54.7 (2.9)	54.4 (2.8)	0.22
Race, *n* (%)	89 (17.2)	49 (19)	40 (16)	0.25	67 (14)	37 (15)	30 (12)	0.08
Mexican American	99 (19.2)	49 (19)	50 (19)		108 (22)	51 (21)	57 (24)	
Other Hispanic	209 (40.5)	110 (43)	99 (39)		212 (44)	102 (42)	110 (46)	
Non–Hispanic White	44 (8.5)	21 (8)	23 (9)		35 (7)	13 (5)	22 (9)	
Non–Hispanic Black	75 (14.5)	29 (11)	46 (18)		63 (13)	40 (17)	23 (10)	
Other race								
Education, *n* (%)	146 (28.3)	84 (33)	62 (24)	< 0.01*	137 (28)	70 (29)	67 (28)	0.56
College or above	146 (28.3)	83 (33)	63 (24)		138 (29)	67 (28)	71 (29)	
College or associate degree	126 (24.4)	52 (20)	74 (29)		97 (20)	49 (20)	48 (20)	
High school	98 (19.0)	39 (15)	59 (23)		113 (23)	57 (24)	56 (23)	
Other (below high school)								
Body mass index (kg/m^2^)	29.0 (5.6)	30.3 (6.1)	27.7 (4.7)	< 0.01*	28.8 (5.7)	30.4 (6.4)	27.2 (4.5)	< 0.01*
Arthritis, *n* (%)	62 (12.0)	26 (10)	36 (14)	0.22	101 (21)	57 (24)	44 (18)	0.18
Cancer, *n* (%)	9 (1.7)	5 (2)	4 (2)	1	37 (8)	19 (8)	18 (7)	1
Diabetes, *n* (%)	51 (9.9)	30 (12)	21 (8)	0.16	76 (16)	50 (21)	26 (11)	< 0.01*
Moderate activity (min/week)	31.2 (58.3)	29.7 (58.9)	32.7 (57.9)	0.56	31.9 (62.3)	34.7 (70.0)	29.1 (53.4)	0.33
Sedentary activity (min/day)	390.8 (203.7)	399.2 (209.8)	382.4 (197.5)	0.35	385.9 (198.9)	391.3 (203.6)	380.5 (194.2)	0.55
Energy intake (kcal/day)	2341 (926)	2348 (872)	2333 (978)	0.86	2272 (896)	2287 (863)	2257 (930)	0.71
Protein intake (g/day)	93.2 (39.1)	94.1 (39.4)	92.4 (38.9)	0.63	91.9 (40.5)	91.4 (37.6)	92.4 (43.3)	0.8
Carbohydrate intake (g/day)	277.6 (121.1)	279.7 (111.2)	275.4 (130.3)	0.69	268.9 (116.9)	271.8 (117.3)	266.0 (116.6)	0.58
Fibre intake (g/day)	18.7 (10.8)	19.2 (11.1)	18.1 (10.4)	0.23	19.6 (11.6)	20.4 (12.1)	18.8 (11.0)	0.13
Fat intake (g/day)	87.9 (41.1)	89.9 (40.9)	85.8 (41.5)	0.27	85.3 (41.4)	86.8 (40.8)	83.8 (42.1)	0.43
Total cholesterol (mg/dL)	201.2 (42.3)	202.6 (42.6)	199.8 (42.0)	0.45	195.0 (43.3)	193.7 (42.8)	196.4 (43.9)	0.49
High‐density lipoprotein cholesterol (mg/dL)	46.6 (12.4)	43.5 (11.0)	49.6 (13.0)	< 0.01*	48.3 (14.3)	44.9 (11.7)	51.6 (15.8)	< 0.01*
Haemoglobin (mg/L)	15.1 (1.3)	14.9 (1.4)	15.2 (1.2)	0.04*	14.8 (1.4)	14.6 (1.4)	15.0 (1.3)	< 0.01*
Serum albumin (g/L)	4.38 (0.29)	4.37 (0.29)	4.39 (0.28)	0.45	4.28 (0.36)	4.27 (0.30)	4.28 (0.40)	0.64
Total testosterone (ng/dL)	400.1 (164.5)	274.4 (64.7)	525.9 (135.3)	< 0.01*	403.2 (171.1)	272.8 (68.8)	534.2 (140.3)	< 0.01*
Total testosterone cut‐off (ng/dL)	378.2	—	—	—	378.64	—	—	—
Sex hormone binding globulin (nmol/L)	37.98 (18.11);	26.68 (9.47);	47.33 (18.24);	< 0.01*	44.64 (23.19);	32.85 (13.72); *n* = 109	55.58 (24.79); *n* = 118	< 0.01*
	n = 252	*n* = 114	*n* = 138		*n* = 227			
Handgrip strength (kg)	46.8 (6.9)	46.8 (7.0)	45.8 (6.8)	0.08	43.4 (7.1)	43.0 (6.9)	43.7 (7.3)	0.25
Appendicular lean soft tissue index (kg/m^2^)	8.96 (1.38)	9.20 (1.45)	8.72 (1.28)	< 0.01*	8.77 (1.38)	9.03 (1.48)	8.51 (1.23)	< 0.01*

* Indicates significance.

### 3.1 Association of Higher Total Testosterone With Handgrip Strength and Lean Soft Tissue

The interaction analysis between higher total testosterone and age group was not statistically significant in the fully adjusted models for both ALSTI (*p* = 0.14) and HGS (*p* = 0.96). A negative association was found only for ALSTI in the unadjusted Model 1 (*b* = −0.31, 95% CI −0.41 to −0.21, *p* < 0.01). When the entire cohort was examined, higher testosterone presented a linear positive association with ALSTI in Model 3 (*b* = 0.11, 95% CI 0.02–0.21, *p* = 0.02). Despite the nonsignificant interaction terms, age‐stratified analyses were conducted given the a priori hypothesis that testosterone–muscle relationships may differ across middle‐age decades.

In Model 2, in adults aged 40–49 and 50–59 years, higher total testosterone was associated with lower ALSTI in both age groups (40–49 years: *b* = −0.52, *p* < 0.01; 50–59 years: *b* = −0.31, *p* < 0.01), but not with HGS (40–49 years: *b* = 0.74, *p* = 0.25; 50–59 years: *b* = −0.48, *p* = 0.08). The negative associations in Model 2 attenuated and changed direction after additional adjustment for arthritis, diabetes and cancer in Model 3. In the fully adjusted Model 3, a positive association was found between higher total testosterone and HGS in adults aged 50–59 years (*b* = 1.35, 95% CI 0.08–2.62, *p* = 0.04), while this association with ALSTI remained unchanged (*b* = 0.12, 95% CI −0.02 to 0.25, *p* = 0.09). In those aged 40–49 years, in the fully adjusted models, both HGS (*b* = −0.20, 95% CI −1.41 to 1.01, *p* = 0.75) and ALSTI (*b* = 0.08, 95% CI −0.05 to 0.22, *p* = 0.24) exhibited a nonsignificant association (Table 3).

**TABLE 3:** Association of higher total testosterone with handgrip strength or appendicular lean soft tissue index.

Aged 40–59 years									
	Unadjusted			Model 2			Model 3		
Outcomes	*p*	*b*	95% CI	*p*	*b*	95% CI	*p*	*b*	95% CI
Handgrip strength	0.70	−0.17	−1.06 to 0.72	0.13	0.67	−0.21 to 1.55	0.13	0.68	−0.20 to 1.55
Appendicular lean soft tissue index	< 0.01*	−0.50	−0.67 to −0.33	0.03*	0.11	0.01–0.21	0.02*	0.11	0.02–0.21

Aged 40–49 years									
	Unadjusted			Model 2			Model 3		
Outcomes	*p*	*b*	95% CI	*p*	*b*	95% CI	*p*	*b*	95% CI
Handgrip strength	0.08	−1.06	−2.25 to 0.14	0.84	−0.12	−1.34 to 1.09	0.75	−0.20	−1.41 to 1.01
Appendicular lean soft tissue index	< 0.01*	−0.47	−0.71 to −0.24	0.30	0.07	−0.07 to 0.21	0.24	0.08	−0.05 to 0.22

Aged 50–59 years									
	Unadjusted			Model 2			Model 3		
Outcomes	*p*	*b*	95% CI	*p*	*b*	95% CI	*p*	*b*	95% CI
Handgrip strength	0.25	0.74	−0.53 to 2.01	0.04*	1.36	0.07–2.66	0.04*	1.35	0.08–2.62
Appendicular lean soft tissue index	< 0.01*	−0.52	−0.76 to −0.27	0.09	0.12	−0.02 to 0.26	0.09	0.12	−0.02 to 0.25

Age group interaction w higher testosterone									
	Unadjusted			Model 2			Model 3		
Outcomes	*p*	*b*	95% CI	*p*	*b*	95% CI	*p*	*b*	95% CI
Handgrip strength	0.08	−0.48	−1.01 to 0.06	0.95	−0.02	−0.56 to 0.53	0.96	−0.01	−0.56 to 0.53
Appendicular lean soft tissue index	< 0.01*	−0.31	−0.41 to −0.21	0.22	0.04	−0.02 to 0.10	0.14	0.04	−0.02 to 0.10

*Note:* Model 2: adjusted for age, body mass index, race and education. Model 3: adjusted for Model 2 and arthritis, cancer and diabetes.

* Indicates significance.

When applying the guidelines cut‐off for total testosterone deficiency in hypogonadism, we found that normal versus total testosterone suspected deficiency had a positive association with ALSTI in the fully adjusted model in the whole cohort (*b* = 0.10, 95% CI 0.00–0.19, *p* = 0.049), whereas no link was found with HGS (*b* = 0.75, 95% CI −0.14 to 1.63, *p* = 0.10). No associations were found for the 40‐ to 49‐year‐old and 50‐ to 59‐year‐old groups, nor when age and normal testosterone interacted (Table S1). In addition, above deficient total testosterone versus total testosterone deficiency was associated with increased ALSTI (*b* = 0.20, 95% CI 0.05–0.34, *p* < 0.01) in the entire cohort and those aged 50–59 years (*b* = 0.21, 95% CI 0.01–0.42, *p* < 0.01) (Table S2). For normal versus total testosterone deficiency, in the fully adjusted models, a positive association was shown only with increased ALSTI (*b* = 0.23, 95% CI 0.08–0.38, *p* < 0.01) in the entire cohort, in those aged 40–49 years (*b* = 0.25, 95% CI 0.03–0.47, *p* < 0.02), and when higher age interacted with normal total testosterone levels (*b* = 0.14, 95% CI 0.05–0.23, *p* < 0.01) (Table S3).

### 3.2 Associating Odds of Higher Total Testosterone With Higher Handgrip Strength or Higher Lean Soft Tissue

In the entire cohort, lower odds of higher total testosterone and higher ALSTI were found only in the unadjusted Model 1 (OR: 0.61, 95% CI 0.47–0.78, *p* < 0.01); however, no link was found following full adjustments in Models 2 and 3 (Model 3 → OR: 1.35, 95% CI 0.94–1.94, *p* = 0.10). On the contrary, higher odds of higher total testosterone and higher HGS were presented in the fully adjusted models (OR: 1.33, 95% CI 1.02–1.74, *p* = 0.04). In addition, a negative association of higher testosterone levels with higher ALSTI was found (OR: 0.61, 95% CI 0.43–0.86, *p* < 0.01), but not with higher HGS (OR: 0.88, 95% CI 0.63–1.25, *p* = 0.48). Higher total testosterone was not linked to both higher HGS (OR: 1.07, 95% CI 0.74–1.56, *p* = 0.71) or higher ALSTI (OR: 1.18, 95% CI 0.71–1.95, *p* = 0.52), following adjustments in Model 3.

In adults aged 50–59 years, a positive and negative association was found for higher total testosterone with higher HGS and higher ALSTI, respectively, in the unadjusted Model 1 (higher HGS → OR: 1.45, 95% CI 1.02–2.08, *p* = 0.04; higher ALSTI → OR: 0.60, 95% CI 0.42–0.86, *p* < 0.01). When the fully adjusted Model 3 was examined, no association was found between higher total testosterone levels with higher ALSTI (OR: 1.36, 95% CI: 0.80–2.29, *p* = 0.25), however, increased odds were shown regarding higher HGS (OR: 1.73, 95% CI 1.17–2.55, *p* < 0.01). Age interaction logistic regression found no links across models (Table 4).

**TABLE 4:** Odds of higher total testosterone with higher handgrip strength or higher appendicular lean soft tissue index.

Aged 40–59 years									
	Unadjusted			Model 2			Model 3		
Outcomes	*p*	OR	95% CI	*p*	OR	95% CI	*p*	OR	95% CI
Higher handgrip strength	0.51	1.09	0.85–1.39	0.04*	1.32	1.01–1.73	0.04*	1.33	1.02–1.74
Higher appendicular lean soft tissue index	< 0.01*	0.61	0.47–0.78	0.09	1.36	0.95–1.95	0.10	1.35	0.94–1.94

Aged 40–49 years									
	Unadjusted			Model 2			Model 3		
Outcomes	*p*	OR	95% CI	*p*	OR	95% CI	*p*	OR	95% CI
Higher handgrip strength	0.48	0.88	0.63–1.25	0.65	1.09	0.75–1.57	0.71	1.07	0.74–1.56
High appendicular lean soft tissue index	< 0.01*	0.61	0.43–0.86	0.52	1.18	0.71–1.95	0.52	1.18	0.71–1.95

Aged 50–59 years									
	Unadjusted			Model 2			Model 3		
Outcomes	*p*	OR	95% CI	*p*	OR	95% CI	*p*	OR	95% CI
Higher handgrip strength	0.04*	1.45	1.02–2.08	< 0.01*	1.70	1.16–2.49	< 0.01*	1.73	1.17–2.55
Higher appendicular lean soft tissue index	< 0.01*	0.60	0.42–0.86	0.25	1.36	0.81–2.29	0.25	1.36	0.80–2.29

Age group interaction w higher testosterone									
	Unadjusted			Model 2			Model 3		
Outcomes	*p*	OR	95% CI	P	OR	95% CI	*p*	OR	95% CI
Higher handgrip strength	0.87	0.99	0.85–1.15	0.28	1.09	0.93–1.28	0.26	1.10	0.93–1.28
Higher appendicular lean soft tissue index	< 0.01*	0.73	0.62–0.85	0.32	1.12	0.90–1.38	0.29	1.12	0.91–1.39

*Note:* Model 2: adjusted for age, body mass index, race and education. Model 3: adjusted for Model 2 and arthritis, cancer and diabetes.

* Indicates significance.

Normal testosterone was positively associated with higher HGS versus total testosterone suspected deficiency (OR: 1.37, 95% CI 1.05–1.80, *p* = 0.02), but not with higher ALSTI (OR: 1.34, 95% CI 0.93–1.92, *p* = 0.12) in the whole cohort. No associations were found in the 40‐ to 49‐year‐old group; however, a positive association was shown in those 50–59 years of age with higher HGS (OR: 1.53, 95% CI 1.03–2.28, *p* = 0.03) and higher age interacting with normal total testosterone, with higher HGS (OR: 1.26, 95% CI 1.07–1.50, *p* < 0.01) (Table S4). Higher than total testosterone deficiency versus testosterone deficiency exhibited positive odds with higher ALSTI in the whole cohort (OR: 3.31, 95% CI 1.42–7.74, *p* < 0.01), in those aged 50–59 years (OR: 3.31, 95% CI 1.42–7.74, *p* < 0.01), and higher age interacting with higher than total testosterone deficiency (OR: 1.82, 95% CI 1.19–2.78, *p* < 0.01) (Table S5). There was a positive association between normal total testosterone versus total testosterone deficiency for higher ALSTI in the fully adjusted model in the entire cohort (OR: 2.48, 95% CI 1.35–4.57, *p* < 0.01). Similar findings were found in those aged 50–59 years (OR: 3.00, 95% CI 1.25–7.22, *p* = 0.01) and higher age interacting with normal total testosterone levels (OR: 1.77, 95% CI 1.23–2.53, *p* < 0.01) (Table S6).

### 3.3 Sensitivity Analyses Accounting for SHBG

In sensitivity analyses adjusting for SHBG, the direction of the associations between total testosterone and both HGS and ALSTI remained broadly consistent with the primary analyses. These results are presented in Tables S7–S11.

## 4 Discussion

This study provides insights into the age‐stratified association between total serum testosterone and indices of muscle health during midlife, focusing on a life‐stage that often precedes overt sarcopenia. While the interaction between age group and higher total testosterone was not statistically significant, our stratified analyses revealed that the association between higher total testosterone and HGS was more pronounced in men aged 50–59 years, which was not presented in the 40‐ to 49‐year‐old group.

These findings provide useful insight into the age groups most susceptible to the emerging impact of declining testosterone levels and onset of sarcopenia. This pattern may reflect cumulative exposure to declining testosterone levels across midlife, with prolonged deficiency or a progressive decline in testosterone leading to a more pronounced effect on muscle function, as displayed in the cohort of 50‐ to 59‐year‐old men compared with the 40‐ to 49‐year‐old group. Interestingly, the fully adjusted models nullified the association between higher total testosterone and decreased ALSTI in both age groups.

In our sub‐group analysis, the threshold for suspected total testosterone deficiency (< 346 ng/dL) was based on the EAU guidelines, which serve as the primary reference for diagnosing and managing men's hypogonadism in Europe. However, there is ongoing debate regarding the appropriate cut‐off for low testosterone. Some guidelines define a—suspicion—range, typically between 230 and 346 ng/dL, where it remains uncertain whether the total testosterone level strictly meets the criteria for hypogonadism. Therefore, we also considered highly suspicious total testosterone levels that are indicative of hypogonadism as defined by the Society for Endocrinology in the United Kingdom (< 230 ng/dL). By analysing both guideline categories, we assessed how different thresholds impact markers of sarcopenia, providing clinically relevant estimates that align with current diagnostic frameworks rather than cohort‐specific distributions.

A graded relationship was observed between declining total testosterone levels and reduced markers of muscle health, particularly for ALSTI. Compared with individuals with total testosterone deficiency (< 230 ng/dL), those with testosterone levels in the ‘above deficiency’ range (i.e., above the deficiency threshold of 230 ng/dL) demonstrated significantly higher odds of having increased ALSTI in the whole cohort and specifically in men aged 50–59 years. Similarly, normal total testosterone when compared with suspected deficiency (< 346 ng/dL), demonstrated a positive yet weaker association (compared with total testosterone deficient) with ALSTI in the fully adjusted model. Linear regression results further supported this trend: normal testosterone versus suspected deficiency was associated with a modest but significant increase in ALSTI, while above‐deficiency testosterone versus deficiency showed a larger increase. These findings suggest a stepwise decline in muscle mass as testosterone levels fall from normal, to suspected deficiency, to clearly deficient, supporting a graded association within guideline‐defined categories. While large population‐based analyses, including the UK Biobank, have reported associations between testosterone and sarcopenia in populations aged approximately 40–70 years, these findings have been less consistent in men, highlighting potential heterogeneity across populations and analytical approaches [20]. Although HGS showed less consistent associations, it too declined with lower testosterone levels, with normal levels linked to higher HGS in the overall cohort and even more so among men aged 50–59 compared with those with suspected total testosterone deficiency.

Much of the discussion around the clinical effects of hypogonadism largely surrounds markers of sexual health, mental state and fatigue; compromising the most common primary endpoints in clinical trials of TRT in men with hypogonadism. However, testosterone age‐related muscle decline and the clinical consequences of such in the context of sarcopenia are rarely discussed in‐depth, despite the strong clinical and physiological link between testosterone and its anabolic role in the body. Indeed, TRT has been consistently shown to improve muscle mass and muscle strength in hypogonadal men. A recent meta‐analysis of randomised controlled trials including 2043 subjects older than 60 years found that two different preparations of TRT improved muscle strength compared with placebo [21]. In the same meta‐analysis, a meta‐regression revealed baseline serum total testosterone levels were indeed associated with muscle strength improvement. Although our study did not assess physical function markers (e.g., gait speed, chair stand test or timed up‐and‐go), prior research has demonstrated associations between testosterone levels and physical performance measures, such as gait speed and Short Physical Performance Battery scores [22, 23], in adults aged 40–70 years, reinforcing the strong link with overall muscle health. In this context, our findings suggest that clinically interpretable differences in muscle health are already observable during midlife, a stage that precedes the populations typically targeted in interventional trials.

This present analysis highlights the need for a deeper understanding of the relationship between age‐related declines in testosterone levels and markers of muscle health in middle age. Loss of muscle mass is a well‐established symptom of testosterone deficiency; therefore, men in middle age may benefit from undergoing a testosterone panel to determine whether deficiency is contributing to muscle loss. Additionally, further research should explore the differential effects of testosterone on functional health, alongside the roles of oestradiol and dihydrotestosterone during middle‐age. Nevertheless, our results may have relevance in older age, considering that age‐related sarcopenia is prevalent. For instance, in the Swedish MrOS study (*n* = 2908, average age 75.4 years) [24], testosterone was found to be an independent positive predictor of bone mineral density in the total body, total hip, femur trochanter and arm. In addition, mendelian randomisation studies have indicated that genetically determined oestradiol levels are more strongly associated with bone mineral density and fracture risk than genetically determined testosterone levels in men [25]. Finally, greater attention should be given to testosterone carrier proteins, particularly SHBG, as elevated SHBG levels can lead to lower testosterone readings but may be linked to other conditions such as hyperthyroidism or the use of certain medications.

### 4.1 Limitations and Future Directions

While the findings of the present study may provide useful insights for future longitudinal research, they should be interpreted in light of several limitations. First, NHANES is a cross‐sectional survey, which precludes any inference of causal relationships between testosterone levels and markers of muscle health. Second, only single total testosterone measurements were obtained for each participant; prior evidence suggests that up to 30% of men with an initial total testosterone value in the deficient range may have normal levels upon repeat testing [26], potentially leading to misclassification. Third, we were unable to assess tissue‐level androgen action. NHANES does not include measures of androgen receptor expression or polymorphisms, androgen receptor co‐regulators, 5‐α‐reductase activity, dihydrotestosterone or gonadotropins; therefore, individuals with attenuated intracellular androgen signalling despite normal or elevated circulating testosterone could not be identified. However, the use of a ID‐LC‐MS/MS assay represents a methodological strength, ensuring high analytical accuracy and precision of total testosterone measurements, particularly in the lower concentration range, where immunoassay‐based approaches used in other large population studies may be more prone to measurement error [27]. Fourth, although SHBG was available in a subset of participants (less than 50% of our cohort), its incomplete availability limited the feasibility of robust analyses incorporating testosterone across the full cohort. The reduced sample size in SHBG‐adjusted models (*n* = 338 to 479) limited statistical power. Effect estimates remained directionally consistent with the main analyses, suggesting that adjustment for SHBG did not fully account for the observed associations. Consequently, our findings should be interpreted primarily as associations with circulating total testosterone, recognising that discordance between serum levels and intracellular androgen action may attenuate or dilute observed effect sizes. Fifth, although dietary intake was included as a confounding factor, it was assessed using the average of two 24‐h dietary recalls, which may not accurately reflect habitual intake and limits longitudinal interpretation of nutritional patterns. In addition, multiple statistical comparisons were performed without formal correction for multiple testing. Primary inferences focus on fully adjusted models and clinically relevant age‐specific associations.

Taken together, these limitations highlight the need for longitudinal studies integrating repeated hormonal assessments, comprehensive androgen profiling (SHBG and other relevant markers), as well as detailed dietary and functional evaluations, to more robustly characterise the relationship between testosterone and muscle health.

Given the established role of testosterone in anabolic processes, the present findings align with known physiological mechanisms and highlight the importance of maintaining adequate testosterone levels with age to preserve muscle mass and muscle strength. Future research should investigate the potential benefits and risks of TRT in men with testosterone levels in the suspected deficiency range who present with impaired muscle strength or physical performance, a clinically ambiguous group. Longitudinal studies are particularly needed to clarify the temporal nature and specificity of the relationship between declining testosterone, ALSTI, and HGS and to determine whether incorporating ALSTI and muscle strength assessments into diagnostic pathways may enhance the identification and management of hypogonadism. Further characterisation of the roles of testosterone, oestradiol and dihydrotestosterone may also provide additional insight into the hormonal regulation of muscle health.

## 5 Conclusions

The present study found that the relationship between testosterone and markers of sarcopenia (HGS, ALST) may vary by age group. In men aged 50–59 years, higher total testosterone levels were positively associated with higher HGS, but not LST, while in the 40‐ to 49‐year age group, no links between variables were found. Additionally, applying guideline‐based cut‐off values for testosterone deficiency further confirmed the relationship between lower total testosterone levels and declines in both ALSTI and HGS among men with both suspected and highly suspicious total testosterone deficiency. These findings suggest that the impact of testosterone on functional muscle health may be more pronounced with age, possibly due to prolonged exposure to declining testosterone levels alongside muscle health, benefiting those with increased total testosterone levels.

## Ethics Statement

The authors of this manuscript certify that they comply with the ethical guidelines for authorship and publishing in the Journal of Cachexia, Sarcopenia and Muscle [28].

## Conflicts of Interest

C.M.P.: speaking engagements or advisory board participation for Abbott Nutrition, Nestle Health Science, Nutricia and Novo Nordisk. All other authors declare no conflicts of interest. J.A.B.: consultant for Abbott Nutrition.

## Supporting information


**Table S1:** Association of normal vs. testosterone insufficiency total testosterone based on the European Association of Urology with handgrip strength or appendicular lean soft tissue index.

**Table S2:** Association of higher than deficiency total testosterone vs. testosterone deficiency based on the European Association of Urology with handgrip strength or appendicular lean soft tissue index.

**Table S3:** Association of normal total testosterone vs. testosterone deficiency based on the European Association of Urology with handgrip strength or appendicular lean soft tissue index.

**Table S4:** Odds of normal vs. testosterone insufficiency based on the European Association of Urology with higher handgrip strength or higher appendicular lean soft tissue index.

**Table S5:** Odds of higher than deficiency total testosterone vs. testosterone deficiency based on the European Association of Urology with higher handgrip strength or higher appendicular lean soft tissue index.

**Table S6:** Odds of normal total testosterone vs. testosterone deficiency based on the European Association of Urology with higher handgrip strength or higher appendicular lean soft tissue index.

**Table S7:** Association of higher testosterone with handgrip strength or appendicular lean soft tissue index accounting for sex hormone binding globulin.

**Table S8:** Odds of higher total testosterone with higher handgrip strength or higher appendicular lean soft tissue index accounting for sex hormone binding globulin.

**Table S9:** Association of normal total testosterone vs. testosterone deficiency based on the European Association of Urology with handgrip strength or appendicular lean soft tissue index accounting for sex hormone binding globulin.

**Table S10:** Association of higher than deficiency total testosterone vs. testosterone deficiency based on the European Association of Urology with handgrip strength or appendicular lean soft tissue index accounting for sex hormone binding globulin.

**Table S11:** Association of normal vs. testosterone insufficiency total testosterone based on the European Association of Urology with handgrip strength or appendicular lean soft tissue index accounting for sex hormone binding globulin.
