# Supplementary material for: Association of Testosterone With Lean Soft Tissue and Handgrip Strength Across Middle‐Aged Men
Source: J Cachexia Sarcopenia Muscle. 2026 Jul 7;17(4):e70329. doi: 10.1002/jcsm.70329 (PMC13341951; doi:10.1002/jcsm.70329)
Supplement: Supplementary file 1 — Table S1: Association of normal vs. testosterone insufficiency total testosterone based on the European Association of Urology with handgrip strength or appendicular lean soft tissue index. [file JCSM-17-e70329-s001.docx]

**Table S1.** Association of normal vs. testosterone insufficiency total testosterone based on the European Association of Urology with handgrip strength or appendicular lean soft tissue index.

| **Aged 40-59 years** | | | | | | | | | |
| --- | --- | --- | --- | --- | --- | --- | --- | --- | --- |
|  | **Unadjusted** | | | **Model 2** | | | **Model 3** | | |
| **Outcomes** | **p** | **b** | **95%CI** | **p** | **b** | **95%CI** | **p** | **b** | **95%CI** |
| Handgrip strength | 0.78 | -0.13 | -1.03 – 0.77 | 0.10 | 0.75 | -0.14 – 1.64 | 0.10 | 0.75 | -0.14 – 1.63 |
| Appendicular lean soft tissue index | <0.01* | -0.51 | -0.68 – -0.34 | 0.05 | 0.10 | -0.002 – 0.19 | 0.049* | 0.10 | 0.000 – 0.19 |
| **Aged 40-49 years** | | | | | | | | | |
|  | **Unadjusted** | | | **Model 2** | | | **Model 3** | | |
| **Outcomes** | **p** | **b** | **95%CI** | **p** | **b** | **95%CI** | **p** | **b** | **95%CI** |
| Handgrip strength | 0.23 | -0.74 | -1.94 – 0.47 | 0.73 | 0.21 | -1.01 – 1.44 | 0.87 | 0.10 | -1.13 – 1.33 |
| Appendicular lean soft tissue index | <0.01* | -0.42 | -0.66 – -0.18 | 0.11 | 0.11 | -0.02 – 0.25 | 0.07 | 0.13 | -0.01 – 0.27 |
| **Aged 50-59 years** | | | | | | | | | |
|  | **Unadjusted** | | | **Model 2** | | | **Model 3** | | |
| **Outcomes** | **p** | **b** | **95%CI** | **p** | **b** | **95%CI** | **p** | **b** | **95%CI** |
| Handgrip strength | 0.39 | 0.57 | -0.72 – 1.85 | 0.07 | 1.22 | -0.09 – 2.53 | 0.06 | 1.22 | -0.07 – 2.51 |
| Appendicular lean soft tissue index | <0.01* | -0.60 | -0.85 – -0.36 | 0.43 | 0.06 | -0.08 – 0.20 | 0.47 | 0.05 | -0.09 – 0.19 |
| **Age group interaction w normal testosterone** | | | | | | | | | |
|  | **Unadjusted** | | | **Model 2** | | | **Model 3** | | |
| **Outcomes** | **p** | **b** | **95%CI** | **p** | **b** | **95%CI** | **p** | **b** | **95%CI** |
| Handgrip strength | 0.04* | -0.57 | -1.10 – -0.03 | 0.07 | 0.51 | -0.05 – 1.06 | 0.07 | 0.51 | -0.05 – 1.06 |
| Appendicular lean soft tissue index | <0.01* | 0.33 | -0.43 – -0.23 | 0.07 | 0.06 | -0.004 – 0.12 | 0.07 | 0.06 | -0.01 – 0.12 |

*Indicates significance.
Model 2: adjusted for age, body mass index, race, and education
Model 3: adjusted for Model 2 and arthritis, cancer, and diabetes
