# Supplementary material for: Association of Testosterone With Lean Soft Tissue and Handgrip Strength Across Middle‐Aged Men
Source: J Cachexia Sarcopenia Muscle. 2026 Jul 7;17(4):e70329. doi: 10.1002/jcsm.70329 (PMC13341951; doi:10.1002/jcsm.70329)
Supplement: Supplementary file 2 — Table S2: Association of higher than deficiency total testosterone vs. testosterone deficiency based on the European Association of Urology with handgrip strength or appendicular lean soft tissue index. [file JCSM-17-e70329-s003.docx]

**Table S2.** Association of higher than deficiency total testosterone vs. testosterone deficiency based on the European Association of Urology with handgrip strength or appendicular lean soft tissue index.

| **Aged 40-59 years** | | | | | | | | | |
| --- | --- | --- | --- | --- | --- | --- | --- | --- | --- |
|  | **Unadjusted** | | | **Model 2** | | | **Model 3** | | |
| **Outcomes** | **p** | **b** | **95%CI** | **p** | **b** | **95%CI** | **p** | **b** | **95%CI** |
| Handgrip strength | 0.97 | 0.03 | -1.35 – 1.41 | 0.15 | 0.98 | -0.36 – 2.33 | 0.15 | 0.98 | -0.36 – 2.32 |
| Appendicular lean soft tissue index | <0.01* | -0.59 | -0.86 – -0.33 | 0.01* | 0.20 | 0.05 – 0.34 | <0.01* | 0.20 | 0.05 – 0.34 |
| **Aged 40-49 years** | | | | | | | | | |
|  | **Unadjusted** | | | **Model 2** | | | **Model 3** | | |
| **Outcomes** | **p** | **b** | **95%CI** | **p** | **b** | **95%CI** | **p** | **b** | **95%CI** |
| Handgrip strength | 0.62 | -0.49 | -2.38 – 1.41 | 0.45 | 0.72 | -1.16 – 2.59 | 0.48 | 0.68 | -1.20 – 2.55 |
| Appendicular lean soft tissue index | <0.01* | -0.55 | -0.93 – -0.17 | 0.16 | 0.15 | -0.06 – 0.37 | 0.14 | 0.16 | -0.05 – 0.37 |
| **Aged 50-59 years** | | | | | | | | | |
|  | **Unadjusted** | | | **Model 2** | | | **Model 3** | | |
| **Outcomes** | **p** | **b** | **95%CI** | **p** | **b** | **95%CI** | **p** | **b** | **95%CI** |
| Handgrip strength | 0.70 | 0.38 | -1.56 – 2.31 | 0.26 | 1.11 | -0.84 – 3.07 | 0.27 | 1.09 | -0.83 – 3.01 |
| Appendicular lean soft tissue index | <0.01* | -0.65 | -1.02 – -0.28 | 0.04 | 0.22 | 0.01 – 0.42 | 0.04* | 0.21 | 0.01 – 0.42 |
| **Age group interaction w normal testosterone** | | | | | | | | | |
|  | **Unadjusted** | | | **Model 2** | | | **Model 3** | | |
| **Outcomes** | **p** | **b** | **95%CI** | **p** | **b** | **95%CI** | **p** | **b** | **95%CI** |
| Handgrip strength | <0.01* | -1.38 | -2.04 – -0.73 | 0.30 | 0.41 | -0.37 – 1.19 | 0.31 | 0.40 | -0.37 – 1.17 |
| Appendicular lean soft tissue index | <0.01* | -0.31 | -0.43 – -0.18 | <0.01* | 0.12 | 0.04 – 0.21 | <0.01* | 0.12 | 0.04 – 0.21 |

*Indicates significance.
Model 2: adjusted for age, body mass index, race, and education
Model 3: adjusted for Model 2 and arthritis, cancer, and diabetes
