# Supplementary material for: Association of Testosterone With Lean Soft Tissue and Handgrip Strength Across Middle‐Aged Men
Source: J Cachexia Sarcopenia Muscle. 2026 Jul 7;17(4):e70329. doi: 10.1002/jcsm.70329 (PMC13341951; doi:10.1002/jcsm.70329)
Supplement: Supplementary file 3 — Table S3: Association of normal total testosterone vs. testosterone deficiency based on the European Association of Urology with handgrip strength or appendicular lean soft tissue index. [file JCSM-17-e70329-s007.docx]

**Table S3.** Association of normal total testosterone vs. testosterone deficiency based on the European Association of Urology with handgrip strength or appendicular lean soft tissue index.

| **Aged 40-59 years** | | | | | | | | | |
| --- | --- | --- | --- | --- | --- | --- | --- | --- | --- |
|  | **Unadjusted** | | | **Model 2** | | | **Model 3** | | |
| **Outcomes** | **p** | **b** | **95%CI** | **p** | **b** | **95%CI** | **p** | **b** | **95%CI** |
| Handgrip strength | 0.97 | -0.03 | -1.46 – 1.40 | 0.14 | 1.10 | -0.36 – 2.56 | 0.14 | 1.10 | -0.35 – 2.54 |
| Appendicular lean soft tissue index | <0.01* | -0.74 | -1.01 – -0.48 | <0.01* | 0.23 | 0.07 – 0.38 | <0.01* | 0.23 | 0.08 – 0.38 |
| **Aged 40-49 years** | | | | | | | | | |
|  | **Unadjusted** | | | **Model 2** | | | **Model 3** | | |
| **Outcomes** | **p** | **b** | **95%CI** | **p** | **b** | **95%CI** | **p** | **b** | **95%CI** |
| Handgrip strength | 0.45 | -0.75 | -2.71 – 1.21 | 0.43 | 0.81 | -1.21 – 2.83 | 0.49 | 0.72 | -1.30 – 2.73 |
| Appendicular lean soft tissue index | <0.01* | -0.67 | -1.05 – -0.29 | 0.04* | 0.23 | 0.01 – 0.45 | 0.02* | 0.25 | 0.03 – 0.47 |
| **Aged 50-59 years** | | | | | | | | | |
|  | **Unadjusted** | | | **Model 2** | | | **Model 3** | | |
| **Outcomes** | **p** | **b** | **95%CI** | **p** | **b** | **95%CI** | **p** | **b** | **95%CI** |
| Handgrip strength | 0.58 | 0.57 | -1.46 – 2.60 | 0.23 | 1.32 | -0.82 – 3.46 | 0.23 | 1.28 | -0.82 – 3.38 |
| Appendicular lean soft tissue index | <0.01* | -0.82 | -1.19 – -0.46 | 0.08 | 0.19 | -0.02 – 0.41 | 0.08 | 0.19 | -0.02 – 0.41 |
| **Age group interaction w normal testosterone** | | | | | | | | | |
|  | **Unadjusted** | | | **Model 2** | | | **Model 3** | | |
| **Outcomes** | **p** | **b** | **95%CI** | **p** | **b** | **95%CI** | **p** | **b** | **95%CI** |
| Handgrip strength | 0.01* | -0.96 | -1.70 – -0.22 | 0.16 | 0.61 | -0.25 – 1.47 | 0.17 | 0.60 | -0.25 – 1.45 |
| Appendicular lean soft tissue index | <0.01* | -0.41 | -0.54 – -0.27 | <0.01* | 0.14 | 0.05 – 0.23 | <0.01* | 0.14 | 0.05 – 0.23 |

*Indicates significance.
Model 2: adjusted for age, body mass index, race, and education
Model 3: adjusted for Model 2 and arthritis, cancer, and diabetes
