# Supplementary material for: Association of Testosterone With Lean Soft Tissue and Handgrip Strength Across Middle‐Aged Men
Source: J Cachexia Sarcopenia Muscle. 2026 Jul 7;17(4):e70329. doi: 10.1002/jcsm.70329 (PMC13341951; doi:10.1002/jcsm.70329)
Supplement: Supplementary file 4 — Table S4: Odds of normal vs. testosterone insufficiency based on the European Association of Urology with higher handgrip strength or higher appendicular lean soft tissue index. [file JCSM-17-e70329-s005.docx]

**Table S4.** Odds of normal vs. testosterone insufficiency based on the European Association of Urology with higher handgrip strength or higher appendicular lean soft tissue index.

| **Aged 40-59 years** | | | | | | | | | |
| --- | --- | --- | --- | --- | --- | --- | --- | --- | --- |
|  | **Unadjusted** | | | **Model 2** | | | **Model 3** | | |
| **Outcomes** | **p** | **OR** | **95%CI** | **p** | **OR** | **95%CI** | **p** | **OR** | **95%CI** |
| Higher handgrip strength | 0.40 | 1.11 | 0.87 – 1.43 | 0.02* | 1.37 | 1.05 – 1.79 | 0.02* | 1.37 | 1.05 – 1.80 |
| Higher appendicular lean soft tissue index | <0.01* | 0.62 | 0.48 – 0.80 | 0.11 | 1.35 | 0.94 – 1.94 | 0.12 | 1.34 | 0.93 – 1.92 |
| **Aged 40-49 years** | | | | | | | | | |
|  | **Unadjusted** | | | **Model 2** | | | **Model 3** | | |
| **Outcomes** | **p** | **OR** | **95%CI** | **p** | **OR** | **95%CI** | **p** | **OR** | **95%CI** |
| Higher handgrip strength | 0.66 | 0.92 | 0.65 – 1.31 | 0.49 | 1.14 | 0.79 – 1.65 | 0.56 | 1.12 | 0.77 – 1.63 |
| Higher appendicular lean soft tissue index | 0.06 | 0.72 | 0.51 – 1.02 | 0.16 | 1.45 | 0.87 – 2.41 | 0.15 | 1.45 | 0.87 – 2.43 |
| **Aged 50-59 years** | | | | | | | | | |
|  | **Unadjusted** | | | **Model 2** | | | **Model 3** | | |
| **Outcomes** | **p** | **OR** | **95%CI** | **p** | **OR** | **95%CI** | **p** | **OR** | **95%CI** |
| Higher handgrip strength | 0.15 | 1.30 | 0.91 – 1.87 | 0.04* | 1.52 | 1.03 – 2.23 | 0.03* | 1.53 | 1.03 – 2.28 |
| Higher appendicular lean soft tissue index | <0.01* | 0.55 | 0.38 – 0.79 | 0.51 | 1.20 | 0.70 – 2.03 | 0.53 | 1.19 | 0.70 – 2.02 |
| **Age group interaction w normal testosterone** | | | | | | | | | |
|  | **Unadjusted** | | | **Model 2** | | | **Model 3** | | |
| **Outcomes** | **p** | **OR** | **95%CI** | **p** | **OR** | **95%CI** | **p** | **OR** | **95%CI** |
| Higher handgrip strength | 0.67 | 0.97 | 0.83 – 1.12 | <0.01* | 1.26 | 1.06 – 1.49 | <0.01* | 1.26 | 1.07 – 1.50 |
| Higher appendicular lean soft tissue index | <0.01* | 0.72 | 0.62 – 0.84 | 0.09 | 1.22 | 0.97 – 1.53 | 0.10 | 1.21 | 0.97 – 1.53 |

*Indicates significance.
Model 2: adjusted for age, body mass index, race, and education
Model 3: adjusted for Model 2 and arthritis, cancer, and diabetes
