# Supplementary material for: Association of Testosterone With Lean Soft Tissue and Handgrip Strength Across Middle‐Aged Men
Source: J Cachexia Sarcopenia Muscle. 2026 Jul 7;17(4):e70329. doi: 10.1002/jcsm.70329 (PMC13341951; doi:10.1002/jcsm.70329)
Supplement: Supplementary file 5 — Table S5: Odds of higher than deficiency total testosterone vs. testosterone deficiency based on the European Association of Urology with higher handgrip strength or higher appendicular lean soft tissue index. [file JCSM-17-e70329-s011.docx]

**Table S5.** Odds of higher than deficiency total testosterone vs. testosterone deficiency based on the European Association of Urology with higher handgrip strength or higher appendicular lean soft tissue index.

| **Aged 40-59 years** | | | | | | | | | |
| --- | --- | --- | --- | --- | --- | --- | --- | --- | --- |
|  | **Unadjusted** | | | **Model 2** | | | **Model 3** | | |
| **Outcomes** | **p** | **OR** | **95%CI** | **p** | **OR** | **95%CI** | **p** | **OR** | **95%CI** |
| Higher handgrip strength | 0.55 | 0.85 | 0.49 – 1.46 | 0.84 | 0.94 | 0.53 – 1.68 | 0.83 | 0.94 | 0.52 – 1.69 |
| Higher appendicular lean soft tissue index | 0.46 | 0.82 | 0.48 – 1.40 | <0.01* | 3.47 | 1.50 – 8.03 | <0.01* | 3.31 | 1.42 – 7.74 |
| **Aged 40-49 years** | | | | | | | | | |
|  | **Unadjusted** | | | **Model 2** | | | **Model 3** | | |
| **Outcomes** | **p** | **OR** | **95%CI** | **p** | **OR** | **95%CI** | **p** | **OR** | **95%CI** |
| Higher handgrip strength | 0.81 | 0.94 | 0.54 – 1.63 | 0.53 | 1.21 | 0.68 – 2.15 | 0.52 | 1.21 | 0.68 – 2.17 |
| High appendicular lean soft tissue index | 0.22 | 0.71 | 0.40 – 1.23 | 0.24 | 1.64 | 0.72 – 3.69 | 0.21 | 1.68 | 0.74 – 3.82 |
| **Aged 50-59 years** | | | | | | | | | |
|  | **Unadjusted** | | | **Model 2** | | | **Model 3** | | |
| **Outcomes** | **p** | **OR** | **95%CI** | **p** | **OR** | **95%CI** | **p** | **OR** | **95%CI** |
| Higher handgrip strength | 0.55 | 0.85 | 0.49 – 1.46 | 0.84 | 0.94 | 0.53 – 1.68 | 0.83 | 0.94 | 0.52 – 1.69 |
| Higher appendicular lean soft tissue index | 0.46 | 0.82 | 0.48 – 1.40 | <0.01* | 3.47 | 1.50 – 8.03 | <0.01* | 3.31 | 1.42 – 7.74 |
| **Age group interaction w higher than deficiency testosterone** | | | | | | | | | |
|  | **Unadjusted** | | | **Model 2** | | | **Model 3** | | |
| **Outcomes** | **p** | **OR** | **95%CI** | **p** | **OR** | **95%CI** | **p** | **OR** | **95%CI** |
| Higher handgrip strength | 0.55 | 0.92 | 0.70 – 1.21 | 0.84 | 0.97 | 0.73 – 1.29 | 0.83 | 0.97 | 0.72 – 1.30 |
| Higher appendicular lean soft tissue index | 0.46 | 0.90 | 0.69 – 1.18 | <0.01* | 1.86 | 1.22 – 2.83 | <0.01* | 1.82 | 1.19 – 2.78 |

*Indicates significance.
Model 2: adjusted for age, body mass index, race, and education
Model 3: adjusted for Model 2 and arthritis, cancer, and diabetes
