# Supplementary material for: Association of Testosterone With Lean Soft Tissue and Handgrip Strength Across Middle‐Aged Men
Source: J Cachexia Sarcopenia Muscle. 2026 Jul 7;17(4):e70329. doi: 10.1002/jcsm.70329 (PMC13341951; doi:10.1002/jcsm.70329)
Supplement: Supplementary file 6 — Table S6: Odds of normal total testosterone vs. testosterone deficiency based on the European Association of Urology with higher handgrip strength or higher appendicular lean soft tissue index. [file JCSM-17-e70329-s009.docx]

**Table S6.** Odds of normal total testosterone vs. testosterone deficiency based on the European Association of Urology with higher handgrip strength or higher appendicular lean soft tissue index.

| **Aged 40-59 years** | | | | | | | | | |
| --- | --- | --- | --- | --- | --- | --- | --- | --- | --- |
|  | **Unadjusted** | | | **Model 2** | | | **Model 3** | | |
| **Outcomes** | **p** | **OR** | **95%CI** | **p** | **OR** | **95%CI** | **p** | **OR** | **95%CI** |
| Higher handgrip strength | 0.84 | 0.96 | 0.65 – 1.43 | 0.49 | 1.16 | 0.76 – 1.78 | 0.49 | 1.17 | 0.76 – 1.80 |
| Higher appendicular lean soft tissue index | 0.03* | 0.64 | 0.43 – 0.96 | <0.01* | 2.48 | 1.36 – 4.55 | <0.01* | 2.48 | 1.35 – 4.57 |
| **Aged 40-49 years** | | | | | | | | | |
|  | **Unadjusted** | | | **Model 2** | | | **Model 3** | | |
| **Outcomes** | **p** | **OR** | **95%CI** | **p** | **OR** | **95%CI** | **p** | **OR** | **95%CI** |
| Higher handgrip strength | 0.71 | 0.90 | 0.51 – 1.59 | 0.63 | 1.16 | 0.63 – 2.14 | 0.65 | 1.15 | 0.62 – 2.13 |
| High appendicular lean soft tissue index | 0.11 | 0.63 | 0.35 – 1.11 | 0.20 | 1.76 | 0.75 – 4.17 | 0.15 | 1.91 | 0.80 – 4.56 |
| **Aged 50-59 years** | | | | | | | | | |
|  | **Unadjusted** | | | **Model 2** | | | **Model 3** | | |
| **Outcomes** | **p** | **OR** | **95%CI** | **p** | **OR** | **95%CI** | **p** | **OR** | **95%CI** |
| Higher handgrip strength | 0.98 | 1.01 | 0.58 – 1.76 | 0.75 | 1.11 | 0.60 – 2.05 | 0.77 | 1.10 | 0.59 – 2.06 |
| Higher appendicular lean soft tissue index | 0.13 | 0.65 | 0.37 – 1.13 | 0.01* | 3.15 | 1.32 – 7.51 | 0.01* | 3.00 | 1.25 – 7.22 |
| **Age group interaction w normal testosterone** | | | | | | | | | |
|  | **Unadjusted** | | | **Model 2** | | | **Model 3** | | |
| **Outcomes** | **p** | **OR** | **95%CI** | **p** | **OR** | **95%CI** | **p** | **OR** | **95%CI** |
| Higher handgrip strength | 0.08 | 0.83 | 0.67 – 1.02 | 0.37 | 1.12 | 0.87 – 1.45 | 0.38 | 1.12 | 0.87 – 1.45 |
| Higher appendicular lean soft tissue index | <0.01* | 0.74 | 0.60 – 0.91 | <0.01* | 1.77 | 1.24 – 2.53 | <0.01* | 1.77 | 1.23 – 2.53 |

*Indicates significance.
Model 2: adjusted for age, body mass index, race, and education
Model 3: adjusted for Model 2 and arthritis, cancer, and diabetes
