# Supplementary material for: Association of Testosterone With Lean Soft Tissue and Handgrip Strength Across Middle‐Aged Men
Source: J Cachexia Sarcopenia Muscle. 2026 Jul 7;17(4):e70329. doi: 10.1002/jcsm.70329 (PMC13341951; doi:10.1002/jcsm.70329)
Supplement: Supplementary file 7 — Table S7: Association of higher testosterone with handgrip strength or appendicular lean soft tissue index accounting for sex hormone binding globulin. [file JCSM-17-e70329-s006.docx]

**Table S7.** Association of higher testosterone with handgrip strength or appendicular lean soft tissue index accounting for sex hormone binding globulin.

|  | **Aged 40-59 years** | | |
| --- | --- | --- | --- |
| **Outcomes** | **p** | **b** | **95%CI** |
| Handgrip strength | 0.26 | 0.83 | 0.71 – 2.26 |
| Appendicular lean soft tissue index | 0.02* | 0.19 | 0.03 – 0.35 |
|  | **Aged 40-49 years** | | |
| **Outcomes** | **p** | **b** | **95%CI** |
| Handgrip strength | 0.44 | -0.76 | -2.73 – 1.19 |
| Appendicular lean soft tissue index | 0.06 | 0.11 | -0.01 – 0.22 |
|  | **Aged 50-59 years** | | |
| **Outcomes** | **p** | **b** | **95%CI** |
| Handgrip strength | 0.02* | 1.21 | 0.33 – 4.52 |
| Appendicular lean soft tissue index | 0.13 | 0.08 | -0.02 – 0.19 |

Adjusted for age, body mass index, race, education, arthritis, cancer, diabetes, and sex hormone binding globulin.
