# Supplementary material for: Association of Testosterone With Lean Soft Tissue and Handgrip Strength Across Middle‐Aged Men
Source: J Cachexia Sarcopenia Muscle. 2026 Jul 7;17(4):e70329. doi: 10.1002/jcsm.70329 (PMC13341951; doi:10.1002/jcsm.70329)
Supplement: Supplementary file 8 — Table S8: Odds of higher total testosterone with higher handgrip strength or higher appendicular lean soft tissue index accounting for sex hormone binding globulin. [file JCSM-17-e70329-s008.docx]

**Table S8.** Odds of higher total testosterone with higher handgrip strength or higher appendicular lean soft tissue index accounting for sex hormone binding globulin.

|  | **Aged 40-59 years** | | |
| --- | --- | --- | --- |
| **Outcomes** | **p** | **OR** | **95%CI** |
| Handgrip strength | 0.18 | 1.35 | 0.87 – 2.09 |
| Appendicular lean soft tissue index | 0.03* | 2.05 | 1.10 – 3.83 |
|  | **Aged 40-49 years** | | |
| **Outcomes** | **p** | **OR** | **95%CI** |
| Handgrip strength | 0.50 | 0.80 | 0.51 – 1.55 |
| Appendicular lean soft tissue index | 0.13 | 1.99 | 0.82 – 4.80 |
|  | **Aged 50-59 years** | | |
| **Outcomes** | **p** | **OR** | **95%CI** |
| Handgrip strength | 0.02* | 2.21 | 1.15 – 4.24 |
| Appendicular lean soft tissue index | 0.11 | 2.16 | 0.85 – 5.48 |

Adjusted for age, body mass index, race, education, arthritis, cancer, diabetes, and sex hormone binding globulin.
