# Supplementary material for: Association of Testosterone With Lean Soft Tissue and Handgrip Strength Across Middle‐Aged Men
Source: J Cachexia Sarcopenia Muscle. 2026 Jul 7;17(4):e70329. doi: 10.1002/jcsm.70329 (PMC13341951; doi:10.1002/jcsm.70329)
Supplement: Supplementary file 9 — Table S9: Association of normal total testosterone vs. testosterone deficiency based on the European Association of Urology with handgrip strength or appendicular lean soft tissue index accounting for sex hormone binding globulin. [file JCSM-17-e70329-s004.docx]

**Table S9.** Association of normal total testosterone vs. testosterone deficiency based on the European Association of Urology with handgrip strength or appendicular lean soft tissue index accounting for sex hormone binding globulin.

|  | **Aged 40-59 years (n = 338)** | | |
| --- | --- | --- | --- |
| **Outcomes** | **p** | **b** | **95%CI** |
| Handgrip strength | 0.84 | 0.25 | -2.14 – 2.64 |
| Appendicular lean soft tissue index | 0.08 | 0.22 | -0.02 – 0.46 |
|  | **Aged 40-49 years (n = 175)** | | |
| **Outcomes** | **p** | **b** | **95%CI** |
| Handgrip strength | 0.29 | -1.84 | -5.23 – 1.55 |
| Appendicular lean soft tissue index | 0.22 | 0.24 | -0.14 – 0.62 |
|  | **Aged 50-59 years (n = 163)** | | |
| **Outcomes** | **p** | **b** | **95%CI** |
| Handgrip strength | 0.37 | 1.56 | -1.87 – 4.99 |
| Appendicular lean soft tissue index | 0.17 | 0.23 | -0.10 – 0.56 |

Adjusted for age, body mass index, race, education, arthritis, cancer, diabetes, and sex hormone binding globulin.
