# Supplementary material for: Association of Testosterone With Lean Soft Tissue and Handgrip Strength Across Middle‐Aged Men
Source: J Cachexia Sarcopenia Muscle. 2026 Jul 7;17(4):e70329. doi: 10.1002/jcsm.70329 (PMC13341951; doi:10.1002/jcsm.70329)
Supplement: Supplementary file 10 — Table S10: Association of higher than deficiency total testosterone vs. testosterone deficiency based on the European Association of Urology with handgrip strength or appendicular lean soft tissue index accounting for sex hormone binding globulin. [file JCSM-17-e70329-s002.docx]

**Table S10.** Association of higher than deficiency total testosterone vs. testosterone deficiency based on the European Association of Urology with handgrip strength or appendicular lean soft tissue index accounting for sex hormone binding globulin.

|  | **Aged 40-59 years (n = 479)** | | |
| --- | --- | --- | --- |
| **Outcomes** | **p** | **b** | **95%CI** |
| Handgrip strength | 0.92 | -0.11 | -2.20 – 1.98 |
| Appendicular lean soft tissue index | 0.35 | 0.11 | -0.12 – 0.34 |
|  | **Aged 40-49 years (n = 252)** | | |
| **Outcomes** | **p** | **b** | **95%CI** |
| Handgrip strength | 0.26 | -1.73 | -4.73 – 1.28 |
| Appendicular lean soft tissue index | 0.70 | 0.07 | -0.30 – 0.44 |
|  | **Aged 50-59 years (n = 227)** | | |
| **Outcomes** | **p** | **b** | **95%CI** |
| Handgrip strength | 0.62 | 0.74 | -2.20 – 3.67 |
| Appendicular lean soft tissue index | 0.30 | 0.16 | -0.14 – 0.46 |

Adjusted for age, body mass index, race, education, arthritis, cancer, diabetes, and sex hormone binding globulin.
