# Supplementary material for: Association of Testosterone With Lean Soft Tissue and Handgrip Strength Across Middle‐Aged Men
Source: J Cachexia Sarcopenia Muscle. 2026 Jul 7;17(4):e70329. doi: 10.1002/jcsm.70329 (PMC13341951; doi:10.1002/jcsm.70329)
Supplement: Supplementary file 11 — Table S11: Association of normal vs. testosterone insufficiency total testosterone based on the European Association of Urology with handgrip strength or appendicular lean soft tissue index accounting for sex hormone binding globulin. [file JCSM-17-e70329-s010.docx]

**Table S11.** Association of normal vs. testosterone insufficiency total testosterone based on the European Association of Urology with handgrip strength or appendicular lean soft tissue index accounting for sex hormone binding globulin.

|  | **Aged 40-59 years (n = 479)** | | |
| --- | --- | --- | --- |
| **Outcomes** | **p** | **b** | **95%CI** |
| Handgrip strength | 0.26 | 0.83 | -0.60 – 2.25 |
| Appendicular lean soft tissue index | 0.09 | 0.14 | -0.02 – 0.30 |
|  | **Aged 40-49 years (n = 252)** | | |
| **Outcomes** | **p** | **b** | **95%CI** |
| Handgrip strength | 0.66 | -0.45 | -2.44 – 1.54 |
| Appendicular lean soft tissue index | 0.10 | 0.21 | -0.04 – 0.45 |
|  | **Aged 50-59 years (n = 227)** | | |
| **Outcomes** | **p** | **b** | **95%CI** |
| Handgrip strength | 0.08 | 1.90 | -0.19 – 3.99 |
| Appendicular lean soft tissue index | 0.45 | 0.08 | -0.13 – 0.30 |

Adjusted for age, body mass index, race, education, arthritis, cancer, diabetes, and sex hormone binding globulin.
